# Supplementary material for: The Construction and Exploration of a Comprehensive MicroRNA Centered Regulatory Network in Foxtail Millet (Setaria italica L.)
Source: Front Plant Sci. 2022 May 6;13:848474. doi: 10.3389/fpls.2022.848474 (PMC9121102; doi:10.3389/fpls.2022.848474)
Supplement: Supplementary file 4 [file Data_Sheet_4.zip › Sit-miRNovel2_Seita.4G000900.1_377_TPlot.pdf]

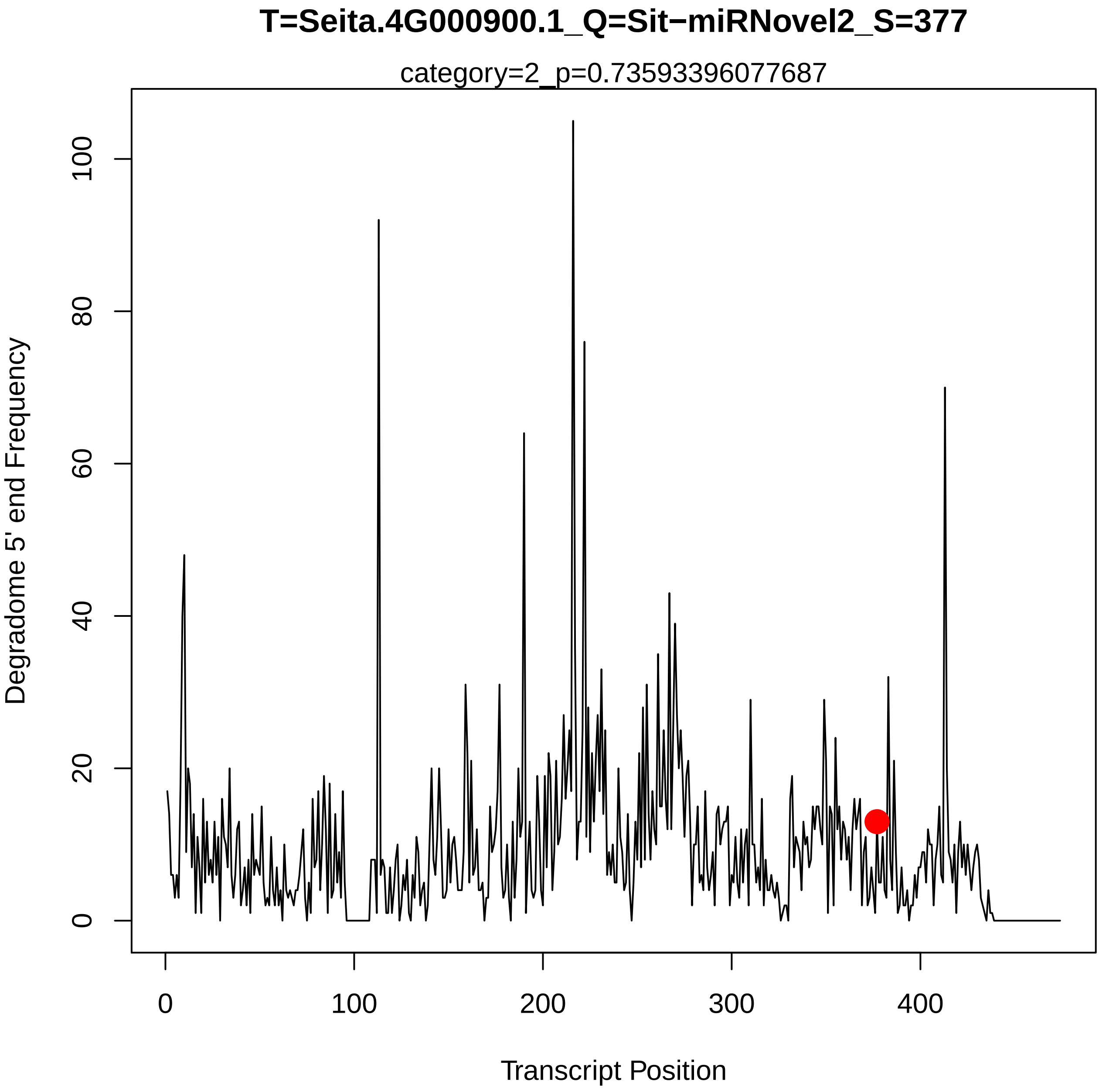

T=Seita.4G000900.1_Q=Sit−miRNovel2_S=377
category=2_p=0.73593396077687
Degradome 5' end Frequency
Transcript Position
0
20
40
60
80
100
0
100
200
300
400
